# Tactics and Economics of Wildlife Oral Rabies Vaccination, Canada and the United States

**DOI:** 10.3201/eid1508.081061

**Published:** 2009-08

**Authors:** Ray T. Sterner, Martin I. Meltzer, Stephanie A. Shwiff, Dennis Slate

**Affiliations:** US Department of Agriculture, Fort Collins, Colorado, USA (R.T. Sterner, S.A. Shwiff); Centers for Disease Control and Prevention, Atlanta, Georgia, USA (M.I. Meltzer); US Department of Agriculture, Concord, New Hampshire, USA (D. Slate)

**Keywords:** Coyote, economics, fox, North America, oral rabies vaccination, rabies, raccoon, viruses, zoonoses, synopsis

## Abstract

Progressive elimination of rabies in wildlife has been a general strategy in Canada and the United States; common campaign tactics are trap–vaccinate–release (TVR), point infection control (PIC), and oral rabies vaccination (ORV). TVR and PIC are labor intensive and the most expensive tactics per unit area (≈$616/km^2^ [in 2008 Can$, converted from the reported $450/km^2^ in 1991 Can$] and ≈$612/km^2^ [$500/km^2^ in 1999 Can$], respectively), but these tactics have proven crucial to elimination of raccoon rabies in Canada and to maintenance of ORV zones for preventing the spread of raccoon rabies in the United States. Economic assessments have shown that during rabies epizootics, costs of human postexposure prophylaxis, pet vaccination, public health, and animal control spike. Modeling studies, involving diverse assumptions, have shown that ORV programs can be cost-efficient and yield benefit:cost ratios >1.0.

## Abstract 2

Economic assessments and modeling studies suggest that these programs yield cost savings and public health benefits.

Rabies continues to pose major public health concerns in Canada and the United States (*1*–*5*). Effective pet vaccination programs have controlled rabies in domestic dogs (*Canis familiaris*) in both countries, but rabies persists in wildlife reservoirs. In 2007, a total of 6,776 cases in wildlife were reported for the contiguous United States (*1*).

Oral rabies vaccination (ORV) is an evolving rabies control technology for use in wildlife (*6*). It involves distribution of baits containing orally immunogenic vaccines onto the landscape, thereby targeting wildlife to establish population immunity and prevent spread or eliminate specific rabies variants (*6*).

We reviewed the literature on ORV programs and economics in Canada and the United States. The first use of ORV sought to control rabies in red foxes (*Vulpes vulpes*) in Switzerland; subsequent programs were reported throughout much of western Europe (*7*,*8*). Switzerland, France, Belgium, and Luxembourg were deemed free of the red fox variant by 2001 (*8*).

## ORV in Ontario, Canada

### Arctic Fox–Variant Rabies in Red Foxes

**Figure 1:**
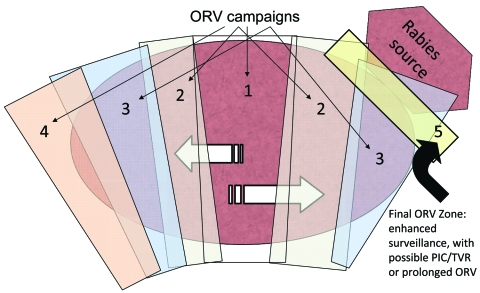
Expanding-wedge tactic with progressive elimination (*9*). Numbers represent successive oral rabies vaccination (ORV) zones. Potential savings are assumed for the area of progressive elimination, southern Ontario Province. The rectangle bordering the rabies source (i.e., 5) highlights an area of enhanced surveillance, possible point infection control (PIC) activities, trap–vaccinate–release (TVR) activities, or an ORV zone intended to deter future reemergence of the virus.

During 1989–1995, ORV was used in Ontario to progressively eliminate arctic fox (*Alopex lagopus*)–variant rabies that had spilled into (i.e., had been transmitted to another species) red foxes and spread southward (*9*). Each year ORV baits were distributed in southern Ontario (≈20 baits/km^2^, from aircraft or by hand, over 8,850–29,590 km^2^). The strategy was termed progressive elimination and resembled an expanding ORV wedge, which started near the center of the outbreak and expanded during successive years (Figure 1).

Within 5 years of program initiation, reported cases of rabid foxes declined from 203 cases/year to 4 cases/year in the baited areas (*9*,*10*). Spillover cases from red foxes to striped skunks (*Mephitis mephitis*) and livestock dropped from preepizootic (30-year) means of >36 and >42, respectively, to 0 by 1997 (*9*). Since 2003, only 13 cases of the variant in red foxes have been reported; these continue to be addressed by using focused control and enhanced surveillance (i.e., increased public health monitoring, examination of road-killed target animals, and rabies analyses of samples from trappers) (D. Donovan, Ontario Ministry of Natural Resources, pers. comm.).

### Rabies in Raccoons and Skunks

During 1987–1991, to reduce spillover of rabies from red foxes to urban raccoons (*Procyon lotor*) and skunks, trap–vaccinate–release (TVR; capture live, vaccinate parenterally, and release on site) was integrated into ORV campaigns in the Toronto area (*10*). TVR was part of the red fox ORV program because Evelyn-Rokitnicki-Abelseth oral rabies vaccine is not immunogenic in skunks and raccoons (*6*,*9*). Live traps were set (20–75 traps/km^2^) in a 60-km^2^ portion of the city, and 66,168 ORV baits were distributed by hand in natural areas (20–40 baits/km^2^). Of sampled foxes, 46%–80% had biomarkers from baits, and only 1 rabid fox was found during 1987–1992 (*10*). A recent update of ORV baiting in Toronto stated that 332,257 baits had been distributed during 1989–1999, and only 5 rabid foxes were found during 1990–2006 (*11*).

**Figure 2:**
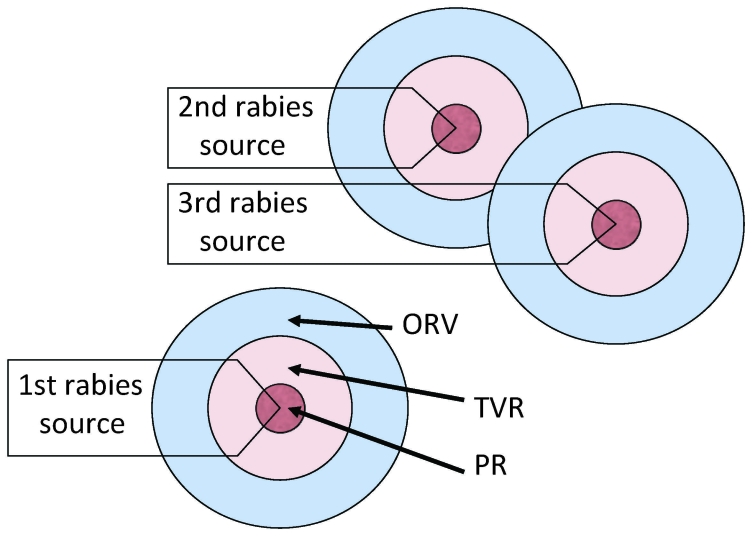
Point infection control (PIC) tactic. Concentric rings around the location of a rabid animal represent vector population reduction (PR), trap–vaccinate–release (TVR), and ORV zones (*12*). Each new source leads to repeated, overlapping ORV, TVR, and PR rings. Potential savings are assumed within the zones and for assumed distances beyond the zones.

During 1999–2000, the raccoon variant of rabies was confirmed near Brockville, Ontario (*12*). To eliminate raccoon-variant rabies from the province, a point infection control (PIC) tactic, which integrated population reduction (PR; sometimes referred to as culling or depopulation), TVR, and ORV, was implemented (*12*). The initial PIC operation included concentric zones, each consisting of 1) an inner 5-km PR zone, 2) a middle 5-km TVR zone, and 3) an outer 8–15-km ORV zone (Figure 2). Additional PIC or modified PIC (no PR) operations were centered on newly discovered rabid raccoons (≈40).

Mean raccoon densities in PR zones dropped from 5.1–7.1/km^2^ before to 0.6–1.1/km^2^ after PIC operations. However, within 1 year, >37 more cases of raccoon-variant rabies occurred in the PIC regions (*12*). Intensive PIC was begun again and eliminated the variant from Ontario. Subsequently, to reduce the chances of raccoon-variant rabies recurring in southern Ontario, enhanced surveillance and annual ORV was conducted along the border of Ontario and New York (D. Donovan, pers. comm.). Elimination of raccoon rabies from Wolfe Island at the mouth of the St. Lawrence River using similar tactics was recently reported (*13*).

## ORV in the United States

### Canine-Variant Rabies in Coyotes in Southern Texas

**Figure 3:**
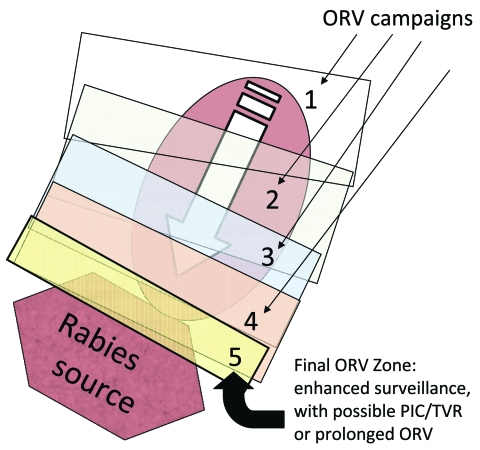
Collapsed-bands tactic with progressive elimination (*17*). Numbers represent successive oral rabies vaccination (ORV) zones that attempt to collapse the baited area, exclude virus incursion outside, and lead to a maintenance zone that prevents reintroduction of the disease after the current population matures and vaccination effects are lost. Potential savings are assumed to occur within the ORV areas and for assumed distances beyond the zone. The rectangle bordering the rabies source (i.e., 5) highlights an area of enhanced surveillance, possible point infection control (PIC) activities, trap–vaccinate–release (TVR) activities, or an ORV zone intended to deter future reemergence of the virus.

During 1988–1994, a canine-variant of rabies described in Mexico was confirmed in 163 domestic dogs and 296 coyotes from 18 counties in southern Texas (*14*–*16*). In 1995, to prevent the northward spread of this variant, ORV baits (9–27 baits/km^2^) were distributed in an arc-shaped band over a 24-county area (39,850 km^2^) ≈200 km north of Laredo (*16*). During 1996–2003, annual baiting continued; ≈9.35 million baits were distributed onto ≈741,766 km^2^ (*17*). Gradually, baits were distributed farther south, toward the Rio Grande River, in subsequent years, thereby collapsing the rabies-infected area (Figure 3). To protect livestock, coyotes were also removed from portions of the ORV zone during these years, but the effect of PR relative to ORV was not assessed (*18*,*19*). PR is considered an important component of many rabies-control models (*20*).

After 1 year of baiting, the mean rate of canine-variant cases at the leading edge of the epizootic area was 2.8/10,000 km^2^. This rate was similar to that of the preepizootic period and suggestive that the northward spread of the epizootic had ceased (*16*). Subsequent surveillance showed a gradual decline in cases from 122 in 1995 to 0 in 2004 (*17*). Currently, to maintain an immune buffer and prevent canine rabies from reemerging in southern Texas, this program baits an ORV zone 30–65 km wide along the international border each year (E. Oertli, Texas Department of State Health Services, pers. comm.).

### Gray Fox–Variant Rabies in West-Central Texas

**Figure 4:**
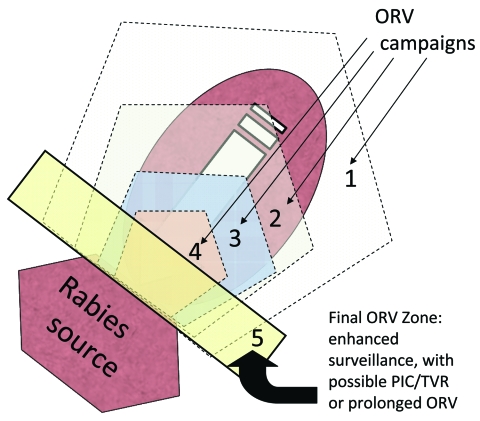
Purse string–like tactic with progressive elimination (*17*). Numbers represent successive oral rabies vaccination (ORV) zones that attempt to roughly encircle and shrink the baited area, exclude virus incursion from outside, and lead to a maintenance zone that prevents reintroduction of the disease after the current population matures and vaccination effects are lost. Potential savings are assumed to occur within the ORV areas and for assumed distances beyond the outer zone. The rectangle bordering the rabies source (i.e., 5) highlights an area of enhanced surveillance, possible point infection control (PIC)/trap–vaccinate–release (TVR) activities, or an ORV zone intended to deter future reemergence of the virus.

During 1988–1994, a total of 283 gray foxes (*Urocyon cinereoargenteus*) and 241 other domestic and wild animals in west-central Texas were confirmed positive for a unique rabies variant typically found in gray foxes (*17*). This outbreak was spatially distinct from the outbreak of canine rabies in southern Texas. To control this epizootic, during 1995–2009 (and ongoing), ORV (29–39 baits/km^2^) was conducted annually by encircling the epizootic area using ≈32-km–wide ORV strips; an added 16- to 24-km vaccination buffer of ORV baits was created along the northern and eastern edges of the rabies-variant area; this tactic has been referred to as a purse string–like tactic (i.e., encircle and shrink) (*17*; Figure 4). An area of ≈350,000 km^2^ was baited annually. Evidence of bait biomarkers and positive rabies virus neutralizing antibody titers was found for 39% and 62% of foxes, respectively, sampled from the ORV zone, confirming that numerous foxes had been vaccinated.

In 2007, new cases of gray fox rabies occurred northwestward along the Pecos River and in west-central Texas. To prevent further spread of this variant, ORV was used (E. Oertli, pers. comm.). The rabies-control goal has not changed from one of containment and elimination of the gray fox variant from Texas. However, in light of recent surveillance, the anticipated strategy of establishing and maintaining an ORV zone along the Rio Grande River to prevent potential reemergence from Mexico has been delayed and is being refined to include prolonged enhanced surveillance as a key factor in allocating resources and gauging success (E. Oertli, pers. comm.).

### Raccoon-Variant Rabies in the Eastern United States

**Figure 5:**
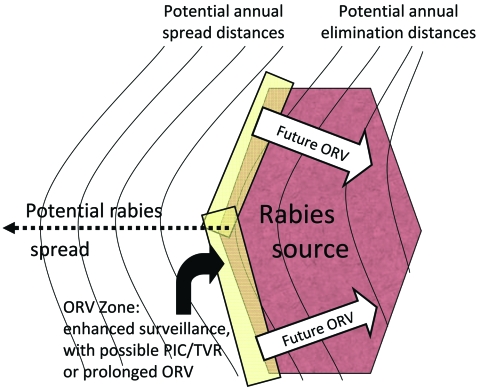
Oral rabies vaccination (ORV) preventive spread or elimination tactic with eventual progressive elimination (*22*). The ORV zone of vaccinated animals is intended to prevent spread of the disease beyond the ORV zone; potential elimination is assumed to result from successive baiting campaigns into the infected area. Potential savings are assumed beyond the ORV zone (or within the zone, if elimination is possible); disease spread rates, final distances of infectious impacts, and durations of ORV bait distributions ultimately determine the magnitude of potential savings. PIC, point infection control activities; TVR, trap–vaccinate–release activities.

The National Rabies Management Program began in 1997 and coordinates ORV and related wildlife rabies–control activities in the United States (*21*,*22*). One of its priorities is to prevent the spread of raccoon-variant rabies into uninfected areas, particularly west of its current distribution along the Appalachian Ridge (*22*). The Program integrates natural terrain features (e.g., rivers, lakes, and poor habitat along mountain ridges) with ORV zones (baited at 50–75 baits/km^2^) to create a 40–50 km zone of vaccinated raccoons to help prevent the spread of the virus (Figure 5).

During 1997–2007, the ORV zone was expanded from parts of Ohio to encompass parts of 8 states (i.e., Ohio, Pennsylvania, West Virginia, Virginia, Tennessee, North Carolina, Georgia, and Alabama) along the Appalachian Ridge. A total of 58 bait distributions (usually 1/year) totaling ≈41,018,800 baits and covering ≈530,825 km^2^ (range of 28,660 km^2^ to 84,225 km^2^/distribution) have characterized this effort as of 2007 (R. Hale, US Department of Agriculture, pers. comm.). On the basis of rates of spread of 30–60 km/year in the Mid-Atlantic states before 1997 (*22*–*24*), ORV is viewed as having slowed movement of the virus and, with contingency actions to eliminate some dispersed cases, prevented westward spread of rabies among raccoons. Relatively low and variable vaccination rates have been found, despite the use of relatively high bait densities (50–100/km^2^). Estimated raccoon vaccination rates, based solely on the index of rabies virus neutralizing antibody response, range from 10% to 55% (*22*). The need to vaccinate annually is dictated mainly by high death rates for juveniles and a relatively young age structure for raccoons in North America; juveniles often account for 50% of raccoon populations (*22*). Still, enhanced and public health surveillance indicate that areas west of the Appalachian Ridge remain free of raccoon-variant rabies (*1*,*22*,*23*).

To maintain the integrity of the Appalachian Ridge ORV zone, contingency actions have been needed. In 2004, emergency ORV baiting and TVR were used in northeast Ohio between the established ORV zone and the eastern suburbs of Cleveland (*25*). TVR of >300 raccoons and multiple ORV distributions occurred in this contingency action. This ORV zone had been widened earlier because of encroachment of rabid raccoons from Pennsylvania (*26*).

Other contingency actions unrelated to the westward spread of raccoon rabies have also been implemented. In 2004, an ORV zone created near the Cape Cod Canal to prevent spread of raccoon-variant rabies onto Cape Cod, Massachusetts, was breached, and raccoon-variant rabies spread rapidly throughout the peninsula (T. Algeo, US Department of Agriculture, pers. comm.). Currently, ORV is used twice a year (spring and fall) in the eastern half of the Cape, and baiting is moved gradually westward until an ORV zone can be reestablished along the Cape Cod Canal (J.C. Martin, Tufts Cummings School of Veterinary Medicine, pers. comm.). Additionally, to prevent raccoon rabies from reemerging in southern Ontario, ORV baiting for raccoon-variant rabies continues in northern New York. Confirmed positive raccoon-variant cases in southern Quebec have led to extensive PIC and ORV campaigns to prevent the disease from reaching Montreal. Together, these events and contingency actions illustrate the challenges posed by raccoon rabies, the importance of enhanced surveillance, plus the need to anticipate unexpected contingency actions and their related costs as a component of ORV campaigns.

## Rabies-related Costs

Several studies have documented the costs associated with wildlife-rabies epizootics (*27**–**31*; see Technical Appendix 1). Costs have been adjusted for inflation to 2008 US$ or Can$. A raccoon-variant rabies epizootic in the early 1990s in Hunterdon and Warren Counties, New Jersey, more than doubled rabies-related control costs from $6.67/county resident at $591/km^2^ ($4.05/county resident and $359/km^2^, US$ in 1990) to $16.13/county resident at $1,503/km^2^ ($9.79/county resident at $913/km^2^, US$ in 1990) (*27*).

In Massachusetts, a multiyear study found that the median cost of postexposure prophylaxis (PEP) was $3,356/patient ($2,376/patient; range $1,038–$4,447, US$ in 1995); 69% of the cost was for biologics (*28*). Numbers of PEP administrations increased 26-fold, from 1.7/100,000 residents in 1991 to 45/100,000 residents in 1995 (*28*). Estimates for Connecticut were similar (*29*).

A raccoon-variant epizootic in New York State began in 1991, and the resultant rate of PEP administrations ranged from the equivalent of 24 to 34/100,000 residents (no preepizootic estimates of PEP given) (*30*). During 1998–1999, the mean PEP cost was $1,501/person treated ($1,136/person, US$ in 1998; biologics and administration), equivalent to between $36,024 and $51,034/100,000 residents ($27,264 and $38,624/100,000, US$ in 1998); New York City’s population is excluded from these estimates. This lower cost compared with that for Massachusetts (*28*) and Connecticut (*29*) may be the result of local public health department coordination of PEP administrations in New York State (*30*).

Recently (1998–2002), rabies exposure costs were estimated at $4,066/patient ($3,688/patient, US$ in 2005) in southern California (*31*). Average direct (biologics, medical costs) and indirect costs (travel to physicians, day care for medical appointments) were estimated at $2,827/patient and $1,239/patient, respectively ($2,564/patient and $1,124/patient, US$ in 2005).

## ORV Program Costs

Bait costs and detailed descriptions of the areas baited, which allowed computations of unit area expenses, are available in Table 1 and Technical Appendix 2. ORV programs in Canada and the United States have lasted from >1 for some to >11 years for others and have often required integration of contingency actions (Table 1). The most expensive tactics have been labor-intensive PIC and TVR, but their effectiveness is crucial to maintaining the overall integrity of certain ORV campaigns (*10**–**13**,**22**,**25*). PIC programs have been reported to cost $612/km^2^ ($500/km^2^, Can$ in 1999); costs reported for 3 PIC operations for raccoons totaled $469,247 ($363,100, Can$ in 1999; *12*). TVR costs have ranged from $616/km^2^ to $1,573/km^2^ ($450/km^2^ to $1,150/km^2^, Can$ in 1991; Table 1).

**Table 1:** Major oral rabies vaccination campaigns, Canada and the United States

Country and reference	Strategy or tactic	Duration, y	Target species	Unit bait cost*	Target bait Density, no./km^2^	ORV, TVR, or PIC area, km^2^/y†	Cost/km^2^‡
Canada							
(*9*)	ORV progressive elimination	>7§	Red fox	Not reported	18–20	8,850–31,460	No estimate
(*10*)	TVR	5§	Skunk, raccoon, red fox	>$2.00 (Can$ 1991)	20/den fox only	60	$450–$1,150 (Can$ in 1991)
(*12*)	PIC	>1§	Raccoon	>$2.00 (Can$)	70	225 PR, 485 TVR, 1,200 ORV	$500 (Can$ in 1999)
United States§							
D. Slate, unpub. data (2007)	ORV zone (Appalachian Ridge)	>1§	Raccoon	$1.22 (US$)	50–75	28,659–84,225	$108 (US$ in 2007)
(*26*)	ORV zone (Ohio–Pennsylvania border)	4§	Raccoon	$1.37–$1.52 (US$)	75	3,872–6,497	$153; range $102–$262 (US$ in 1999)¶
(*17*)	ORV progressive elimination	>9§	Coyote	Not reported	19–27	38,850	$42 (US$ in 2004)#
(*17*)	ORV progressive elimination	>8§	Gray fox	Not reported	27–39	56,202	$42 (US$ in 2004)#

*Unless otherwise noted, costs are in Can$ or US$. No discounting for inflation was used; this article and Technical Appendix 2 provide inflation-corrected costs in 2008 Can$ or US$. †Components of reported areas differ according to tactic and strategy. Oral rabies vaccination (ORV) entails topographic areas at which baits are distributed at target densities over landscape. Trap–vaccinate–release (TVR) involves relatively limited topographic areas of intense live trapping and parenteral vaccination of captured animals. Point infection control (PIC) involves successive concentric rings of population reduction, TVR and ORV; the concentric rings become distorted if subsequent rabid animals are caught within these rings. New concentric rings are now formed according to these occurrences. Additionally, depending on habitat or location of urban centers, ORV may be used in a larger strip to create an added ORV preventive zone. ‡Most cost estimates include purchase of baits, aircraft, fuel, and equipment but often omit accurate labor charges. §Surveillance, TVR, PIC, or ORV bait distributions continue at present; therefore, current duration and areas baited differ from those reported. According to Foroutan et al. (*26*), ORV baitings continue as part of the National ORV Program (Slate et al. [*22*]). ¶According to Foroutan et al. (*26*), areas were baited twice each year. In 1997, the first baiting was conducted over a smaller area (1,780 km^2^), and in May 1997 (initial) and June 1999, 2 smaller emergency baitings (in response to a breach in the ORV zone) were conducted, covering <1,701 km^2^. Average costs include a baiting in April 1999, when several tests of bait densities (high) were conducted. #According to Sidwa et al. (*17*), the area baited had shrunk over time because of progressive coyote-variant rabies elimination, but the purse string (gray fox) tactic and ORV-baited area were expanded in 2007 as the gray fox variant spread north along the Pecos River and into southern Texas. The area cost estimate was derived as the quotient of a reported $3.8 million/year program cost and average annual 33,669 km^2^ (dog and coyote) and 56,202 km^2^ (gray fox) bait zones (sum 89,871 km^2^) cited in Technical Appendix 2.

The target species of ORV greatly affects costs, mainly because of species-specific, bait-density requirements. Bait densities for foxes and coyotes have been less than half those for raccoons (Table 1). Thus, gray fox and coyote ORV programs in Texas averaged $48/km^2^ ($42/km^2^, US$ in 2004; Table 1), and raccoon programs in the eastern United States averaged between $111/km^2^ ($108/km^2^, US$ in 2007) and $198/km^2^ ($153/km^2^, US$ in 1999). Cumulative cost of the Appalachian Ridge ORV program has totaled ≈$57 million since its inception in 1997; baits accounted for 72% of the funds expended (Tables 1 and 2).

**Table 2:** Approximate, undiscounted total costs of largest oral rabies vaccine programs, North America, 1989–2004*

Location, target species	Years	Total undiscounted costs, million $	Average undiscounted annual costs, million US$	Reference
Ontario, red foxes	1989–2000	Can$43†	3.5	S.A. Shwiff, unpub. data*
Texas, coyotes and gray foxes	1995–2003	US$34	3.8	(*17*)‡
Appalachian Ridge, raccoons	1997–2007	US$57	5.2	D. Slate, unpub. data§

*Costs are estimates in Can$ or US$ as reported in original publication or as cited by unpublished source. †S.A. Shwiff et al. (unpub. data) based their calculations on certain data presented in *9,32*. ‡Sidwa et al. (*17*) stated that (for both programs combined) average annual costs were $3.8 million. We computed this value as follows: 9 years × $3.8 million = $34 million total (i.e., Sidwa et al. did not clarify what was included in their cost estimate). §D. Slate (2007, unpub. data) provided air, bait, fuel, and staff costs, although some staff hours and fuel costs were omitted for initial campaigns during 1997–2001; a total of 9,394 staff hours, $5,868,262 aircraft costs, $923,481 fuel costs, and $50,187,380 bait costs were reported for 58 campaigns involving the dispensing of 41,018,811 baits over 530,825 km^2^. The software used to determine bait distribution costs was prepared by staff of the United States Department of Agriculture, Animal and Plant Health Inspection Service, Wildlife Services, National Rabies Management Program. After deselecting the bait zones, flight lines were drawn by using the topography (e.g., avoiding water and residential areas) to determine the flight lines and transects. After that had been established, the bait zones were populated with the lines and measured to determine the total length (km). Flight lines determine total flight hours: ([km × 0.539958 nautical miles]/flight speed [knots] = flight hours). Fuel usage is computed as follows: (flight hours × consumption rate [91 gallons/h] × fuel price/gallon = total fuel cost). Costs were also influenced by air transect width, distance to airports for refueling, and end-of-transect turning distance.

Annual costs vary as changes in ORV zones occur, as contingency actions occur, and as ORV programs shift from preventing spread to eliminating variants in given geographic areas. Individual bait prices in the United States range from $1.00 to $1.25 (US$ in 2008, depending on bait type). Because of improved production efficiency, bait prices have decreased slightly during the past 5 years.

## Potential ORV-Induced Savings

One ex post study (actual returns, after the fact) provided detailed estimates of PEP administrations in Ontario during 1956–2000 (*32*). Annual PEP administrations increased from ≈1,000/year during the 1960s and 1970s to >2,000/year during 1982–1993, then decreased to ≈1,000/year again after large-scale ORV campaigns targeting red foxes began in 1989 (*9*,*10*,*32*). Many factors could account for these changes, including revisions of PEP administration guidelines. The initial increase in PEP administrations possibly occurred as a result of fewer adverse effects from use of the new human diploid cell vaccine and stability in numbers of rabies cases (*32*). The latter decrease in PEP administrations was coincident with ORV-caused elimination of arctic fox–variant rabies from southern Ontario (*9*).

## Modeling the Benefits and Costs of ORV

Measured costs of an epizootic of raccoon rabies in New Jersey were used to model the costs and benefits of a hypothetical ORV program (*27*). The model projected net savings for ORV (Table 3) based on the assumptions that the ORV program would require a 2-year campaign and that expenditures to protect human health would remain constant. The model did not allow for reintroduction of rabies or for the potential reemergence of rabies. Benefit:cost ratios (BCRs) related to this hypothetical use of ORV were reported as >2.2 (*27*; Technical Appendix 3, summarizes key principles of benefit:cost modeling).

**Table 3:** Comparison of selected modeling studies that examined the economics of oral rabies vaccination programs*

Reference	Locale, tactics, target species	Type of study, model	Duration modeled, y	Cost and density of vaccine baits; distribution costs†	Results	Comments
(*27*)	2 counties in New Jersey, ORV, raccoon	Benefit:cost, cost data collected from field with hypothetical baiting program	5	$1–$2/bait; 62–200 baits/km^2^; distribution $100/km^2^	Net savings $13.34–$20.78/ county resident (1990 US$); $1,244/km^2^ – $1,939/km^2^	Probably unrealistic: assumed only 2 baitings; no contingency costs; main economic benefit = reduced pet vaccinations
(*33*)‡	Hypothetical 34,447 km^2^-area, expanding circle then maintained barrier zone, raccoon	Benefit:cost of hypothetical baiting program, extensive sensitivity analyses	30	$1.50/ bait; 100 baits/km^2^ (range 40–115); distribution $39/km^2^ (maximim $100/ km^2^)	Net savings of $3.1 million if reduced pet vaccinations included as benefit. Net cost ($6.2 million) if pet vaccinations excluded.	Lack of data required many assumptions; bait density, cost/ bait, and value of pet vaccinations were the most critical elements
(*34*)§¶	Appalachian Ridge area, ORV, raccoon	Benefit:cost model of program to deter westward spread of raccoon rabies	20	$1.30/bait, 75 baits/km^2^ ; aerial distribution $8.62/ km^2^; evaluation $15/km^2^	Net savings $100–$500 million (2000 US$)	Assumed that without ORV, rabies would move 42 or 125 km/y west; distribution costs are low; animal vaccinations are critical component
(*26*)#	Ohio–Pennsylvania, ORV zone (400 km^2^), raccoon	Simulation of individual raccoons + benefit:cost model to prevent westward spread of raccoon rabies	40	$1.47/bait; 3 scenarios of 70, 100, 175 baits/km^2^. Distribution $23.23/km^2^	Net costs (1999 US$; savings recouped 5 km band west of zone)	Complex model showing importance of many biological factors determining potential for success and net savings
(*35*)	Texas, progressive elimination, collapsed bands, coyote	Retrospective benefit:cost model; projected population-based PEP and animal test costs for 20 southern to 232-county expansion area	12	$26.3 million total cost (2006 US$; Texas Department of State Health Services accumulated value)	Net savings $98–$354 million; BCRs of 3.7–13.4; range of savings for 100%, 50% and 25% of PEP and rabies tests in epizootic area.	Simple model showing wide-area expansion. ORV proved cost-efficient if projections were reduced to 7% of the PEP and tests for epizootic counties
S.A. Shwiff, unpub. data	Ontario, progressive elimination, expanded wedge, arctic-fox variant, red fox	Benefit:cost measured costs but had to model savings	12	$77.4 million (2006 Can$) for total ORV	Net savings in 3 of 4 scenarios: reductions in animal rabies testing accounted for most net savings.	Assumed multiple estimates of future rabies-related costs

*No inflation corrections used. ORV, oral rabies vaccination; PEP, postexposure prophylaxis; BCRs, benefit:cost ratios. †Distribution costs exclude cost of bait purchases. US$ except as indicated. ‡For example, Meltzer (*33*) posited a baseline assumption with a distribution cost of $39/km^2^. §Kemere et al. (*34*) assumed that the “… effectiveness of vaccination programs would be validated through surveillance and testing of raccoon populations in the ORV zones … [evaluation cost] also includes educational, promotional, and overhead expenses.” ¶Although Kemere et al. (*34*) did not explicitly allow for contingency costs (to allow for breaches of ORV zones, etc.), they did sensitivity analyses assuming “… the full program costs are used for the entire period instead of dropping to 40% after 5 years.” #Foroutan et al. (*26*) only considered benefits extending up to 5 km west of the ORV zone. A simple extrapolation would suggest that net savings would occur if the calculated benefits were to extend some 100–150 km west of the ORV zone.

Use of ORV to eliminate raccoon rabies from a hypothetical area of 34,447 km^2^ was modeled under 2 scenarios (*33*). Scenario 1 assumed that concentric ORV zones (rings) would expand outward from a center over a 20-year period and that the ORV zone would be maintained for 10 more years to prevent reintroduction. Scenario 2 assumed that the entire area would be baited in the first 2 years and that a ring-shaped ORV zone would be maintained for 28 more years. In the first scenario, inclusion of an expected 20% increase in pet vaccinations (*27*) as a benefit resulted in $3.1 million net savings from ORV; removing pet vaccinations as a savings yielded a net cost of $7.7 million ($6.2 million, US$ in 2000; Table 3). The second scenario yielded no net savings unless the cost of maintaining a containment zone was removed from the model (*33*).

The economics of a large-scale ORV program to prevent the westward spread of raccoon-variant rabies in the eastern United States was modeled and used in planning the current Appalachian Ridge program (*34*). Scenarios assumed that a raccoon-variant rabies epizootic would advance in 40 or 127 km/year (fixed rates) bands to the west of the current leading edge of raccoon-variant rabies along the Appalachian Ridge (*22*). Input variables were as follows: 7% discount rate, 102,650 km^2^ ORV zone, 75 baits/km^2^, $1.63/bait ($1.30/bait, US$ in 2005), $10.78/km^2^ ($8.62/km^2^, US$ in 2000) aerial distribution, and $18.75/km^2^ ($15.00/km^2^, US$ in 2000) post-ORV evaluation. The effect of an epizootic was calculated in terms of unit human population within bands. Results showed that all 8 scenarios, except the 40 km/year spread rate with 20-year fixed baiting costs, yielded BCRs >1.1 and that total estimated net present values of the program were $48–496 million with >$96 million in discounted program costs (*34*). Because of natural geographic features, raccoon population dynamics, and other factors that affect the spatial and temporal spread of rabies, an assumed variable spread of the virus westward would have been more realistic (*25*,*26*). As in previous models (*27*,*33*), estimates of net savings (>50%) for scenarios were enhanced by inclusion of potential pet vaccination costs.

Another model examined specific costs of baiting campaigns for raccoon rabies along the Ohio–Pennsylvania border (*26*). This model incorporated movement and life-cycle data for rabid and nonrabid raccoons. An area of 400 km^2^ with a 10-km ORV zone was assumed to be baited. Benefits were predicted to accrue mainly in a 5-km strip on the west side of the ORV zone. Assumptions about raccoon carrying capacity and percentage ORV vaccination efficiency influenced the rate of rabies spread. This model predicted a net cost for ORV; however, a simple extrapolation implied that net savings would have occurred if the benefits were projected for a 100-km strip west of the ORV zone (*26*).

Ex post modeling was conducted for the ORV campaigns that eliminated red fox–vectored rabies in Ontario. Estimated ORV benefits (PEP + animal rabies tests + livestock indemnity) ranged from $35.4 million to $99.3 million ($35 million to $98 million, Can$ in 2007; total program costs were $78.0 million ($77 million, Can$ in 2007) (S.A. Shwiff, unpub. data). BCRs ranged from 0.49 to 1.36, and outputs implied a lag effect for savings; BCRs were <1.0 during 1990–1992 and >1.0 during 1993–2000.

Recently, an ex post modeling analysis was performed for the 1995–2006 ORV program that eliminated canine-variant rabies from southern Texas (*16*,*17*,*35*). Total expenditures for the ORV program were compared with benefits accrued from likely PEP administrations and animal rabies tests estimated for the 20-county epizootic area and projected to an area involving most of the state. Estimated benefits ranged from $95 million to $369 million ($89 million to $346 million, US$ in 2006); total ORV program costs were reported as $28 million ($26,358,221 US$ in 1995–2006). BCRs ranged from 3.4 to 13.1, depending on assumed incidence of PEP administrations and animal tests (*35*). This study confirmed that 56/100,000 residents received PEP during the epizootic, a high rate for the sparsely populated area of southern Texas where the disease occurred (*35*).

## Conclusions

ORV of wildlife has had positive public health effects. Multiyear campaigns have led to progressive elimination of arctic fox–variant and canine-variant rabies in Ontario and Texas, respectively. PIC, TVR, and ORV zones have prevented raccoon-variant rabies from becoming established in Ontario. Campaigns to contain and eliminate rabies in gray foxes of west-central Texas continue, and spillover of gray fox–variant rabies into coyotes may pose new challenges for preventing the spread of this variant. The ORV zones and contingency actions along the Appalachian Ridge have, thus far, prevented westward spread of raccoon rabies. Habitat alterations to reduce potential carrying capacities of raccoons through local no-feeding regulations and improved refuse management would aid rabies control efforts, but these measures are difficult to implement and enforce. Improved bait-vaccine technology, potentially integrating reproductive inhibitors into TVR campaigns for specific urban raccoon and skunk populations, may improve wildlife rabies elimination.

Rabies campaigns have been relatively expensive. We estimate that >$130 million (combined Can$ and US$) has been spent on ORV programs in North America during the past 10 years. Programs have proved lengthy (typically >5 years), have required enhanced surveillance, and have often required contingency actions to ensure rabies elimination without reintroduction.

Most economic assessments and modeling studies indicate that ORV programs can yield cost savings (*32*–*35*). Regional increases in PEP administrations (and associated public health costs) from 2–4/100,000 before to 24/100,000 (*30*), 45/100,000 (*28*), or 66/100,000 (*27*) residents during or after have been documented for nonbat rabies epizootics. Reduced PEP, epizootic-related pet vaccinations, animal diagnostic tests, public education activities, and other factors represent costs avoided by ORV programs. Direct estimates of wild mammal populations and the relationship of these to numbers of PEP administrations are difficult to obtain; this topic was beyond the scope of our review but needs research.

## Supplementary Material

Technical Appendix 1 Wildlife Rabies-related Costs (Details of Published Studies; No Inflation Corrections Used)

Technical Appendix 2 Correction for Inflation of Selected Costs in Original Publications

Technical Appendix 3 Principles of an Economic Analysis of Oral Rabies Vaccination Programs
