# Supplementary material for: Tactics and Economics of Wildlife Oral Rabies Vaccination, Canada and the United States
Source: Emerg Infect Dis. 2009 Aug;15(8):1176–84. doi: 10.3201/eid1508.081061 (PMC2815952; doi:10.3201/eid1508.081061)
Supplement: Technical Appendix 1 — Wildlife Rabies-related Costs (Details of Published Studies; No Inflation Corrections Used) [file 08-1061_Techapp1-s1.pdf]

Tactics and Economics of Wildlife Oral Rabies Vaccination, Canada and the United States

Technical Appendix 1

Wildlife Rabies-related Costs (Details of Published Studies; No Inflation Corrections Used)

A cost-comparison study examined expenditures for controlling rabies before (1988) and during (1990) a raccoon-variant rabies epizootic in Hunterdon and Warren counties, New Jersey, USA (*1*). This study examined perhaps the most diverse set of costs thus far reported, but these entailed relatively short-term, small-area estimates. The epizootic more than doubled rabies-related control costs, from $4.05 ($359/km^2) to $9.79/county resident ($913/km^2, 1990 US$). Both before and during the epizootic, pet vaccinations were the largest single cost component of rabies expenses; the costs for vaccinations of domestic animals were $337,998/100,000 residents in 1988 and $640,552/100,000 residents in 1990. The next largest category was "other rabies control activities" (e.g., public health, public education) accounting for 11% of costs in 1988 and 13% in 1990. The number of persons receiving postexposure prophylaxis (PEP) increased from 2 in 1988 (1/100,000 residents) to 131 in 1990 (66/100,000 residents). Although the average cost was $555/person treated in 1988 and $1,138/person in 1990, PEPs only accounted for 8% of the rabies-related costs at the peak of the epizootic in New Jersey in 1999.

In Massachusetts, a multiyear study focused on the increased use and cost of PEP during 1991 (1 year before a rabies epizootic) and from 1992 through 1995 of a raccoon rabies epizootic in the state (*2*). The median cost of PEP was $2,376/person (range: $1,038–$4,447; 1995 US$); 69% of the cost was due to biologics. Estimates were similar for Connecticut (*3*). Numbers of PEP administrations increased from 1.7/100,000 residents in 1991 to 45/100,000 residents in 1995 (26-fold increase). Thus, this rabies epizootic increased PEP-related costs by $102,880/100,000 residents.

During the 1990s, New York State reported an epizootic of raccoon-variant rabies (*4*). The number of PEPs given in the state during the epizootic ranged from 2,422 in 1995 to a high of 3,373 in 1997 (no preepizootic numbers reported; New York City (NYC) is excluded from this statewide public health study). These are roughly equivalent to 24 and 34 PEPS per 100,000 residents (calculated using 2000 census count of 10 million state residents, excluding NYC). The mean PEP cost was $1,136/person treated (1998 US$, biologics and administration), equivalent to between $27,264/100,000 and $38,624/100,000 residents. This amount is notably lower than the amount recorded in Massachusetts (*2*), but New York coordinates aspects of PEP (*4*).

Recently (1998–2002), when a skunk rabies epizootic spread from San Luis Obispo to Santa Barbara County, the direct and indirect costs due to rabies exposure in southern California were documented, (*5*). County records documented the medical and public health activities required of 134 patients (equivalent to 4.1/100,000 county resident-years, using 2002 population estimates for 5 years). The public health costs included case investigations and animal control expenses. Telephone interviews of 55 patients who were given PEP provided indirect patient-related expenses related to receiving PEP (e.g., alternative medicine, daycare, travel, time lost from work). The mean total cost of a suspected human rabies exposure was $3,688/patient; average direct (biologics, medical costs) and indirect costs were $2,564/patient and $1,124/patient, respectively (2005 US$).

References

1. Uhaa IJ, Data VM, Sorhage FE, Beckley JW, Roscoe DE, Gorsky RD, et al. Benefits and costs of using an orally absorbed vaccine to control rabies in raccoons. J Am Vet Med Assoc. 1992;201:1873–82. PubMed

2. Kreindel SM, McGill M, Meltzer MI, Rupprecht CE, DeMaria A. The cost of rabies postexposure prophylaxis: one state's experience. Public Health Rep. 1998;113:247–51.

3. Centers for Disease Control and Prevention. Rabies post-exposure prophylaxis—Connecticut, 1990–1994. MMWR Morb Mortal Wkly Rep. 1996;45:232–4. PubMed

4. Chang HG, Eidson M, Noonan-Toly C, Trimarchi CV, Rudd R, Wallace BJ, et al. Public health impact of reemergence of rabies, New York. Emerg Infect Dis. 2002;8:909–13. PubMed

5. Shwiff SA, Sterner RT, Jay-Russell M, Parikh S, Bellomy A, Meltzer MI, et al. Direct and indirect costs of rabies exposure: a retrospective study in southern California (1998–2003). J Wildl Dis. 2007;43:251–7. PubMed
