# Supplementary material for: Tactics and Economics of Wildlife Oral Rabies Vaccination, Canada and the United States
Source: Emerg Infect Dis. 2009 Aug;15(8):1176–84. doi: 10.3201/eid1508.081061 (PMC2815952; doi:10.3201/eid1508.081061)
Supplement: Technical Appendix 2 — Correction for Inflation of Selected Costs in Original Publications [file 08-1061_Techapp2-s2.pdf]

Tactics and Economics of Wildlife Oral Rabies Vaccination, Canada and the United States

Technical Appendix 2

Correction for Inflation of Selected Costs in Original Publications

This Technical Appendix provides selected oral rabies vaccination (ORV), point infection control (PIC), trap–vaccinate–release (TVR), and postexposure propylaxis (PEP) costs for studies cited in the article; published costs are corrected for inflation. The annual Consumer Price Index (CPI %) between 1990 and 2007 for "all goods and services (urban consumers)" was used to derive 2008 values. Bank of Canada (http://www.bankofcanada.ca/en/rates/inflation_calc.html) and the United States Bureau of Labor Statistics (http://www.bls.gov/CPI/#overview) were sources of CPI rates.

Table 1. Annual Consumer Price Index inflation (% change between years)*

Year	Canada	United States
1990	4.8	5.4
1991	5.6	4.2
1992	1.4	3.0
1993	1.9	3.0
1994	0.1	2.6
1995	2.2	2.8
1996	1.5	2.9
1997	1.7	2.3
1998	1.0	1.6
1999	1.8	2.2
2000	2.7	3.4
2001	2.5	2.8
2002	2.2	1.6
2003	2.8	2.3
2004	1.8	2.7
2005	2.2	3.4
2006	2.0	3.2
2007	2.2	2.8
2008	2.3	3.8

*Briefly, costs cited in the original publications were compounded for years subsequent to the originally specified oral rabies vaccination, trap–vaccinate–release, point infection control, and postexposure prophylaxis cost estimates. If no monetary year was provided in the original article, we arbitrarily specified a likely year for the authors' calculations based on 1–2-year publication lags. As of June 25, 2008, currency conversion was as follows: 0.98 Can$ = 1.00 US$; whereas, as of May 5, 2009 1.00 Can$ = 1.19 US$.

Table 2. List of originally cited studies and inflated cost estimates for ORV, TVR, PIC, or PEP variables in 2008 Can$ or US$*

Study and reference	Year or assumed year of original monetary estimate	Current cost (Can$ or US$ in 2008)
ORV/TVR/PIC†		
(*1*)	No estimate	No estimate
(*2*)	1990	TVR $616/km^2 Can$
(*3*)	1999	PIC $605/km^2 Can$
		ORV only $245/km^2 Can$
Slate (prior unpub. data)		ORV $111/km^2 US$
(*4*)	1999	ORV $198/km^2 US$
(*5*)	2004	ORV $48/km^2 US$
PEP‡		
(*6*)	1990	$1,874/patient US$
(*7*)	1995	$3,356/patient US$
(*8*)	1995	1,501/patient US$
(*9*)	2005	$4,066/patient US$

*ORV, oral rabies vaccination; TVR, trap–vaccinate–release; PIC, point infection control; PEP, postexposure prophylaxis.
†Rosatte et al. (2001), Slate (prior unpublished data), and Foroutan et al. (2002) report ORV costs for raccoons (i.e., bait densities of 50–75/km^2; Sidwa et al. (2004) report ORV for coyote and gray fox (i.e., bait densities of 19-39/km^2).
‡Chang et al. (2002) report PEP for New York (i.e., biologics are paid by state); Shwiff et al. (2007) report both direct and indirect costs of PEP.

References

1. MacInnes CD, Smith SM, Tinline RR, Ayers NR, Bachmann P, Ball DGA, et al. Elimination of rabies from red foxes in eastern Ontario. J Wildl Dis. 2001;37:119–32. PubMed

2. Rosatte RC, Power MJ, MacInnes CD, Campbell JB. Trap–vaccinate–release and oral vaccination for rabies control in urban skunks, raccoons and foxes. J Wildl Dis. 1992;28:562–71. PubMed

3. Rosatte R, Donovan D, Allan M, Howes LA, Silver A, Bennett K, et al. Emergency response to raccoon rabies introduction into Ontario. J Wildl Dis. 2001;37:265–79. PubMed

4. Foroutan P, Meltzer MI, Smith KA. Cost of distributing oral raccoon-variant rabies vaccine in Ohio: 1997–2000. J Am Vet Med Assoc. 2002;220:27–32. PubMed DOI: 10.2460/javma.2002.220.27

5. Sidwa TJ, Wilson PJ, Moore GM, Oertli EH, Hicks BN, Rohde RE, et al. Evaluation of oral rabies vaccination programs for control of rabies epizootics in coyotes and gray foxes: 1995–2003. J Am Vet Med Assoc. 2005;227:785–92. PubMed DOI: 10.2460/javma.2005.227.785

6. Uhaa IJ, Data VM, Sorhage FE, Beckley JW, Roscoe DE, Gorsky RD, et al. Benefits and costs of using an orally absorbed vaccine to control rabies in raccoons. J Am Vet Med Assoc. 1992;201:1873–82. PubMed

7. Kreindel SM, McGill M, Meltzer MI, Rupprecht CE, DeMaria A. The cost of rabies postexposure prophylaxis: one state’s experience. Public Health Rep. 1998;113:247–51.

8. Chang HG, Eidson M, Noonan-Toly C, Trimarchi CV, Rudd R, Wallace BJ, et al. Public health impact of reemergence of rabies, New York. Emerg Infect Dis. 2002;8:909–13. PubMed

9. Shwiff SA, Sterner RT, Jay-Russell M, Parikh S, Bellomy A, Meltzer MI, et al. Direct and indirect costs of rabies exposure: a retrospective study in southern California (1998–2003). J Wildl Dis. 2007;43:251–7. PubMed
