# Supplementary material for: Tactics and Economics of Wildlife Oral Rabies Vaccination, Canada and the United States
Source: Emerg Infect Dis. 2009 Aug;15(8):1176–84. doi: 10.3201/eid1508.081061 (PMC2815952; doi:10.3201/eid1508.081061)
Supplement: Technical Appendix 3 — Principles of an Economic Analysis of Oral Rabies Vaccination Programs [file 08-1061_Techapp3-s3.pdf]

Tactics and Economics of Wildlife Oral Rabies Vaccination, Canada and the United States

Technical Appendix 3

Principles of an Economic Analysis of Oral Rabies Vaccination Programs

Rabies, Oral Rabies Vaccination and Use of Benefit:cost Analyses

There are often no human rabies cases directly related to an epizootic of terrestrial rabies. Thus, it is often impossible to conduct a cost-effectiveness analysis—an often-recommended method for public health economics studies, in which the analytic output would be cost per averted case of human rabies (*1*). Thus, published studies have used benefit:cost methods, in which researchers attempt to evaluate all of the benefits and costs from ORV programs in dollar values (*1*,*2*). The general benefit:cost equation below illustrates the basic concept:

Net savings (costs) = value of reduced rabies-related costs –
costs of oral rabies vaccination (ORV) program.

Where: Value of reduced rabies–related costs = (some portion of) costs of rabies epizootic = additional pet vaccinations + additional livestock vaccinations + pet replacements + livestock replacements + additional human preexposure rabies prophylaxis + additional human postexposure prophylaxis (PEP) + costs of treating adverse side effects of additional PEPs + costs of case investigations by public health units + additional laboratory tests + quarantine of suspected rabid animals + public educational materials + potential loss of endangered mammals in areas near epizootics (*3*).

To date, prevention of costs of additional pet vaccinations and PEP have been the main economic impacts of rabies epizootics used to evaluate benefits of ORV programs (*4*–*7*). It is controversial to consider additional pet vaccinations as a cost of rabies epizootic (typically, before a rabies epizootic, vaccination rates in dogs in the United States are between 20% and 50% (*8*). Experience has shown that ORV programs cannot guarantee elimination of all rabies

risks to pets (e.g., ORV zones have been breached, bat rabies is not controlled by current ORV programs). It has been argued that it is potentially a benefit, and not a cost, if pet rabies vaccination rates increase, regardless of cause (S.A. Shwiff, unpub. data). Also, pet replacement costs have never, to our knowledge, actually been measured, and there has yet to be a valuation of a loss of endangered species due to a rabies epizootic.

ORV-Related Program Cost

Costs of an ORV program (often multiyear) can be defined as follows (*3*–*5*):

> Costs of an ORV program per year = (area covered in 1 year × vaccine-bait density per unit area × price of individual vaccine-baits) + distribution costs + costs of injuries/ accidents + costs of contingency plans

Vaccine-baits have, to date, been distributed by fixed-wing aircraft, helicopters, and ground (i.e., personnel either walking or driving). Although accidents and injuries associated with ORV operations have been rare, the potential for these costs remain and must be considered in any economic assessment of ORV. Injuries to employees performing baiting operations are typically covered by some form of preexisting insurance (i.e., costs are prepaid and thus not usually included), but potential public injury or death due to various events remains a possibility. To date, in the United States, only 1 accident involving a citizen's exposure to the live vaccine in ORV baits has occurred. The citizen sued the public health agency responsible for distributing the baits. The court, however, ruled in favor of the public health department, incurring zero cost to the public (*9*).

Analytic Timeline

Rabies epizootics usually last $\geq$2 years and may exhibit cyclic patterns (i.e., number of rabid animals increase and decrease as the animal population changes). The costs of controlling rabies can be considered as having 3 different time periods: preepizootic, during epizootic, and postepizootic. It is possible that during the postepizootic period rabies control costs will not subside to preepizootic levels (*1*). Similarly, an ORV program will typically last $\geq$5 years. Thus, an economic study of ORV programs must incorporate the multiyear aspects of benefits and costs.

Perspective and Discounting

Because a substantial part of the costs of a rabies epizootic and an ORV program are borne by public health entities, it is appropriate that any analyses of an ORV program take a societal perspective as the principal perspective. Furthermore, because rabies epizootics and ORV programs may each take several years, benefits and costs must be appropriately discounted (*1*,*2*,*6*).

Units of Analysis

The economics of ORV are determined by human population density (i.e., increased human population results in increased probability and numbers of PEPs, pet vaccinations, etc.). It has been shown that human population density can impact the number of animals tested for rabies (*10*). Thus, it is recommended that both benefits and costs of ORV programs be calculated in terms of $/unit area (S.A. Shwiff, unpub.data). This allows for ready comparison within a program over time (as populations change), comparison between programs (with possible differences by targeted species and locale), and consideration of targeting and prioritization (by economic criteria).

References

1. Haddix AC, Teustch SM, Corso PA. Prevention effectiveness: a guide to decision analysis and economic evaluation. 2nd ed. Oxford (UK): Oxford University Press; 2002.

2. Zerbe RO, Dively DD. Benefit-cost analysis in theory and practice. New York: HarperCollins College Publishers. 1994. p. 369–94.

3. Sterner RT, Smith GC. Modelling wildlife rabies: transmission, economics and conservation. Biol Conserv. 2006;131:163–79. DOI: 10.1016/j.biocon.2006.05.004

4. Uhaa IJ, Data VM, Sorhage FE, Beckley JW, Roscoe DE, Gorsky RD, et al. Benefits and costs of using an orally absorbed vaccine to control rabies in raccoons. J Am Vet Med Assoc. 1992;201:1873–82. PubMed

5. Kreindel SM, McGill M, Meltzer MI, Rupprecht CE, DeMaria A. The cost of rabies postexposure prophylaxis: one state’s experience. Public Health Rep. 1998;113:247–51.

6. Meltzer MI. Assessing the costs and benefits of an oral vaccine for raccoon rabies: a possible model. Emerg Infect Dis. 1996;2:343–9. PubMed

7. Kemere P, Liddel MK, Evangelou P, Slate D, Osmek S. Economic analysis of a large scale oral vaccination program to control raccoon rabies. In: Clark L, Hone J, Shivik JA, Watkins RA, Vercauteren KC, Yoder JK, editors. Human conflicts with wildlife: economic considerations. Fort Collins (CO): US Department of Agriculture; 2002. p. 109–15.

8. Meltzer MI, Rupprecht CE. A review of the economics of the prevention and control of rabies, part 2: rabies in dogs, livestock and wildlife. PharmacoEconomics. 1998;14:481–98. DOI: 10.2165/00019053-199814050-00003

9. Ohio Court of Claims. Megan Kaso et al. v. Ohio Department of Health, case no. 2001-09036, 2003 May 12. p. 13.

10. Gordon ER, Krebs JW, Rupprecht CR, Real LA, Childs JE, Persistence of elevated rabies prevention costs following post-epizootic declines in rates of rabies among raccoons (*Procyon lotor*). Prev Vet Med. 2005;68:195–222. PubMed DOI: 10.1016/j.prevetmed.2004.12.007
